# Sub-chronic lung inflammation after airway exposures to *Bacillus thuringiensis *biopesticides in mice

**DOI:** 10.1186/1471-2180-10-233

**Published:** 2010-09-03

**Authors:** Kenneth K Barfod, Steen S Poulsen, Maria Hammer, Søren T Larsen

**Affiliations:** 1 National Research Centre for the Working Environment, Copenhagen, Denmark; 2 Statens Serum Institut, Copenhagen Denmark; 3 Department of Biomedical Research, The Panum Institute, University of Copenhagen, Copenhagen, Denmark

## Abstract

**Background:**

The aim of the present study was to assess possible health effects of airway exposures to *Bacillus thuringiensis *(Bt) based biopesticides in mice. Endpoints were lung inflammation evaluated by presence of inflammatory cells in bronchoalveolar lavage fluid (BALF), clearance of bacteria from the lung lumen and histological alterations of the lungs. Hazard identifications of the biopesticides were carried out using intratracheal (i.t.) instillation, followed by an inhalation study. The two commercial biopesticides used were based on the Bt. subspecies *kurstaki *and *israelensis*, respectively. Groups of BALB/c mice were i.t instilled with one bolus (3.5 × 10^5 ^or 3.4 × 10^6 ^colony forming units (CFU) per mouse) of either biopesticide. Control mice were instilled with sterile water. BALFs were collected and the inflammatory cells were counted and differentiated. The BALFs were also subjected to CFU counts.

**Results:**

BALF cytology showed an acute inflammatory response dominated by neutrophils 24 hours after instillation of biopesticide. Four days after instillation, the neutrophil number was normalised and inflammation was dominated by lymphocytes and eosinophils, whereas 70 days after instillation, the inflammation was interstitially located with few inflammatory cells present in the lung lumen.

Half of the instilled mice had remaining CFU recovered from BALF 70 days after exposure. To gain further knowledge with relevance for risk assessment, mice were exposed to aerosols of biopesticide one hour per day for 2 × 5 days. Each mouse received 1.9 × 10^4 ^CFU Bt *israelensis *or 2.3 × 10^3 ^CFU Bt *kurstaki *per exposure. Seventy days after end of the aerosol exposures, 3 out of 17 mice had interstitial lung inflammation. CFU could be recovered from 1 out of 10 mice 70 days after exposure to aerosolised Bt *kurstaki*. Plethysmography showed that inhalation of Bt aerosol did not induce airway irritation.

**Conclusions:**

Repeated low dose aerosol exposures to commercial Bt based biopesticides can induce sub-chronic lung inflammation in mice, which may be the first step in the development of chronic lung diseases. Inhalation of Bt aerosols does not induce airway irritation, which could explain why workers may be less inclined to use a filter mask during the application process, and are thereby less protected from exposure to Bt spores.

## Background

Regarded as harmless to humans, *Bacillus thuringiensis *(Bt) is used worldwide as a commercial biopesticide for the pest control of insects. It is typically used in large spray campaigns on open fields or indoor in green houses [1]. The insecticidal effect is largely due to the characteristic ability to produce specific insect toxins from crystal toxin genes mostly harboured on large plasmids [2]. Bt is a Gram positive, endospore-forming bacterium closely related to the opportunistic human pathogen *Bacillus cereus *[3].

Commercial Bt strains have been isolated from human faecal samples and nasal lavage cultures and elevated human IgE antibody levels have been reported after occupational exposure [4-6]. Most epidemiological and occupational studies on biopesticides have focused on immune responses, infection, food poisoning or other gastro-intestinal symptoms [4,7-9]. The possible long-term effects after repeated pulmonary exposure in humans working with Bt biopesticides have not yet been investigated, although the endospore sizes (1-2 μm in diameter) are within inhalable sizes for humans and mice [10,11]. Long-term effect of biopesticide exposure are not likely to be revealed in longitudinal epidemiological studies, since many green house workers are only temporary employed and therefore may have changed occupation at the time of follow-up.

The purpose of the present study was to explore acute and sub-chronic effects of airway exposure to biopesticides, with focus on airway irritation, lung inflammation and clearance of Bt from the lungs. Initially, dose-response and time-response studies were conducted using i.t. instillations. To simulate occupational exposures, mice were in a subsequent experiment exposed repeatedly by inhalation to aerosolised commercially formulated biopesticides based on Bt *israelensis *or Bt *kurstaki*.

## Methods

### Animals

The exposures were performed on BALB/cJ female mice (Taconic M&B, Ry, Denmark), 6-8 weeks old, body weight 18-22 g. Animals were housed up to 10 animals per cage (425 × 266 × 150 mm) and drinking water and food (Altromin no 1324 Brogaard Denmark) was provided *ad libitum*. Light/dark cycles were at 12 hours and room temperature and relative humidity was kept at 19-22°C and 40-60%, respectively. All protocols were approved by the Danish Animal Experiments Inspectorate.

### Bacterial suspensions and CFU determinations

The bacterial suspensions were prepared from commercially available insecticides Vectobac^® ^(Bt *israelensis*) and Dipel^® ^(Bt *kurstaki*) from Valent Biosciences (Sumitomo Chemical Agro Europe, Lyon, France). The suspensions for aerosol generation and intratracheal instillation were prepared from the formulated products by suspending them in sterile, endotoxin-free water. To reduce viscosity (caused by additives) during the high dose instillations of Dipel^®^, mice in one group (experiment 4, cf. Table 1) received product that was subjected to a washing procedure: the Dipel^® ^was suspended and centrifuged and supernatant discharged. This procedure was repeated twice. The final precipitate was re-suspended in sterile water and adjusted for CFU counts.

**Table 1:** Experimental overview

**Exp.No**.	Aim of experiment	Number of mice	Exposure method	Substance	Time Endpoint	Endpoint	Corresponding figure
1	Validation of Inhalation dose	10	Inhalation (1 hour)	Vectobac^®^	1 h	CFU from total lung homogenate	Figure 1

2	Validation of CFU recovery from BALF	8	Inhalation	Vectobac^®^	1 h	CFU from BALF and lavaged lungtissue	None

3	Dose- response relationship *B.t israelensis*	25	Instillation	Vectobac^®^	24 h	Inflammatory cells in BALF	Figure 2

4	Time- response relationship *B.t israelensis *or *B.t kurstaki*	42	Instillation	Vectobac^® ^or Dipel^® ^(washed)	4 h, 24 h, 4 days	CFU and inflammatory cells in BALF	Figure 3

5	Sub-chronic effects of i.t instillations of *B.t israelensis *or *B.t kurstaki*	20	Instillation	Vectobac^® ^or Dipel^®^	70 days	CFU, Inflammatory cells in BALF, Histology	Figure 4 Figure 5

6	Sub-chronic effects of repeated inhalations of *B.t israelensis *or *B.t kurstaki*	18	Inhalation (Repeated)	Vectobac^® ^or Dipel^®^	70 days	Airway irritation, CFU, Inflammatory cells in BALF, Histology	Figure 5

Mice were exposed to *Bt israelensis *(Vectobac^®^) or *Bt kurstaki *(Dipel^®^) by either intratracheal instillation or inhalation of bacterial suspension. At 4 hours (h), 24 h, 4 days or 70 days after exposure, lungs were lavaged and the bronchoalveolar lavage fluid (BALF) was analysed for content of colony forming units (CFU) and inflammatory cells. Furthermore, histological examination of the lung tissue was performed where specified.

All bacterial morphology and CFU determinations were performed once from two plates of *Bacillus cereus *Selective Agar Base (BCSA) supplemented with *Bacillus cereus *selective supplement and egg yolk emulsion (Scharlau, Barcelona, Spain) after 24 hours of incubation at 30°C.

### Exposures

An overview of the experiments conducted is given in Table 1. In order to reduce non-exposure related variation, the control group and exposure groups were run simultaneously and all mice were handled by the same staff.

### Validation of inhaled dose and CFU recovery from BAL fluids (experiments 1 and 2)

In order to validate the inhaled dose during the aerosol exposure, two groups of 5 mice each were exposed to two different concentrations of Vectobac^® ^for one hour and the lungs were excised at the end of exposure. The theoretically inhaled dose per mouse was compared to the actual deposited dose. The theoretically inhaled dose was calculated as: aerosol concentration × the total volume of inhaled air per mouse during the 60 min exposure period. The aerosol concentration during the exposure was calculated from the CFU determined by Gesamtstaubprobenahme (GSP) filter sampler sampling throughout the exposure (BGI Inc., Waltham, MA, USA). The mean inhaled volume of air during one hour exposure per mouse calculated from the obtained respiration data (respiratory rate (min^-1^) × tidal volume (mL) × 60 min) and was determined to be 2.52 L/hour per mouse. The actual deposited dose was determined by CFU in the total lung homogenate (without a preceding BAL procedure). CFU determinations performed once on BCSA as described above.

In order to compare CFU recovery from total lung homogenate to the CFU recovery from extracted BAL fluid, 8 mice were exposed to Vectobac^® ^via aerosol exposure for 1 hour. BAL was performed on 4 mice and the lungs were excised from all 8 mice and homogenised. BAL fluids, homogenate of lavaged and unlavaged lungs were all plated on BCSA plates for the determination of CFU as described and compared.

The aerosols were also monitored for particle size distribution during exposure by aerodynamic particle sizer (APS-3321, TSI inc., Shoreview, MN, USA), and for real-time particle counts by a Lighthouse 3016 particle counter (LHPC) (Lighthouse Worldwide Solutions, Fremont, CA, USA)

### Intratracheal instillations (experiments 3-5)

The mice were anesthetized before instillation by intra peritoneal injection with Hypnorm^® ^(Veta Pharma Ltd., Leeds, UK) and Dormicum^® ^(Roche AG, Basel, Switzerland). The mice were exposed intra tracheal (i.t.) once with 50 μL volume of inoculum using a flexible polyethylene tube attached to a syringe. The control animals were instilled with 50 μL of sterile pyrogen-free water. Correct insertion of the tube into the trachea was assured by using a modified pneumotachometer (National Research Centre for the Working Environment, Copenhagen, Denmark) [12]. To establish a time-response relationship (experiment 4), 10 mice per dose were exposed by i.t instillations to either 3.4 × 10^6 ^CFU Vectobac^® ^or 3.5 × 10^5 ^CFU Dipel^®^. BAL fluids were collected 4 hours, 24 hours or 4 days post exposure and cells were counted and differentiated as described below.

Subsequently, in order to establish a dose-response relationship (experiment 3), 10 mice per dose was exposed by i.t instillations to a Vectobac^® ^dose of 1.25 × 10^4^, 2 × 10^5^, 4.2 × 10^5 ^or 1.2 × 10^6 ^CFU, respectively. BAL fluids were collected 24 hours post exposure and cells were counted and differentiated as described below.

For the sub-chronic study (experiment 5) the instilled doses were 3.4 × 10^6 ^CFU for Vectobac^® ^and 3.5 × 10^5 ^for Dipel^®^.

### Repeated aerosol inhalations (experiment 6)

Mice (n = 9 per group) were inserted into body plethysmographs that were connected to the exposure chamber. The respiratory parameters were obtained for each mouse from a Fleisch pneumotachograph connected to each plethysmograph that allows continuously monitoring of the parameters [13,14]. The exposures were preceded by a period that allowed the mice to adapt to the plethysmographs. Then, a 15 min. period was used to establish baseline (control) values of the respiratory parameters. This period was followed by a 60 min. exposure period and a 15 min recovery period. Mice were exposed 60 min/day for 5 days per week for two weeks with a two-day break in-between. The dose of 5 × 10^4 ^CFU per mouse per exposure was chosen to mimic occupational exposure [15]. Suspensions of bacteria were delivered from a glass syringe, administered by an infusion pump (New England Medical Instruments Inc., Medway, MA, USA) and via a polyethylene tube connected to a Pitt. No. 1 aerosol generator [16]. The aerosol was mixed through a Vigreaux-column and led to a glass/stainless steel exposure chamber as described [17]. Total flow rate through the chamber was 20 L/min and the air input through the aerosol generator was 14 L/min. The aerosol generator and all related equipments were thoroughly cleaned between exposure sessions. During the aerosol exposures, air samples were collected from the breathing zone of the mice for determination of particle size distribution, real-time particle counts and aerosol CFU concentration. This was done by APS at a flow of 5 L/min, LHPC at 2 L/min and by a filter method GSP at 3.5 L/min. The APS monitored the size distribution of particles in the range from 0.542 to 19.81 μm (aerodynamic diameter) in the exposure chamber. Real time particle counts in the exposure chamber was counted by LHPC in the ranges 0.7-2.0 μm and was used for a real time indicator of aerosol concentration. The GSP samplers were mounted with 0.8 μm polycarbonate filters with airflow of 3.5 L/min. All filters were extracted in 5 mL sterile 0.05% Tween-80 in 0.9% NaCl solution by shaking for 15 min at room temperature (500 rpm) in orbital shaking glass flasks and serial dilutions were made for determination of CFU (see above). Determination of respiratory parameters for assessment of irritation in upper respiratory tract, conducting airways and alveolar region, respectively was performed as thoroughly described [18]. Briefly, three types of effects from the respiratory system can be studied simultaneously:

a) *Sensory irritation*. In humans, chemicals stimulating the trigeminal nerve endings of the upper respiratory tract cause irritation that may increase to burning and painful sensations, termed 'sensory irritation'. Formaldehyde, ammonia and methacrolein are examples of compounds being sensory irritants [18-20]. Sensory irritants decrease the respiratory rate in mice due to a reflex causing a break at the end of the inspiratory phase [21].

b) *Bronchial constriction*. Airflow limitation due to bronchial constriction or inflammation of the conducting airways causes a lengthening of the duration of expiration and thus causes an associated decrease in respiratory rate. To quantify this effect, the airflow rate during expiration is measured.

c) *Pulmonary irritation *is caused by stimulation of vagal nerve endings at the alveolar level [22]. Stimulation of this reflex is characterized by a pause at the end of expiration, which is a specific marker of pulmonary irritation. Ozone is an example of a substance inducing pulmonary irritation [18].

The assessments and quantifications of the respiratory frequency, time of inspiration, time of expiration, time from end of inspiration until the beginning of expiration termed "time of brake", time from end of expiration until beginning of the next inspiration termed "time of pause", tidal volume and mid-expiratory flow rate were performed using the Notocord Hem software (Notocord Systems SA, Croissy-sur-seine, France) as described in details previously [23].

For the comparison of CFU recovered from total lung homogenate to that of CFU recovered from BAL fluid, a pilot inhalation experiment with 8 mice was performed.

### BAL procedure

The BAL procedure was performed as previously described with minor modifications (Larsen *et al*., 2007). Briefly, the lungs were flushed four times with 0.8 mL saline (0.9%) and the recovered fluids were pooled for each mouse. From the BAL fluid of mice that have received bacterial inocula, a 250 μL of total fluid was removed before centrifugation for CFU determination. Cells were counted and differentiated by morphology into neutrophils, lymphocytes, macrophages, epithelial cells and eosinophils. For each sample, 200 cells were differentiated.

### Histopathology

The chest of the mice was opened and a polyethylene tube introduced into the trachea. The polyethylene tube was connected to a syringe containing 4% buffered paraformaldehyde, and the lungs were inflated *in situ *with the fixative to normal size. After 5 minutes the lungs were removed *in toto *and further fixated for at least 24 hours. Tissues were embedded in paraffin in a standardized way (horizontal cut through the hilum regions) and subsequently 7 μm thick slices were cut and stained with haematoxylin/periodic acid Schiff (PAS). The degree of inflammation and morphological changes in the lungs were evaluated blindly by microscopy by two experienced researchers and revaluated in case of discrepancy as described previously [24].

### Statistics

The numbers of inflammatory cells in biopesticide-exposed mice were compared to the control group by means of non-parametric analysis of variance (Kruskall-Wallis). In case of significant difference in the Kruskall-Wallis test, pair-wise comparisons between the water control group and the biopesticide-exposed animals were further analysed using the Mann-Whitney's U-test. Statistical significant difference was accepted at p < 0.05.

## Results

### Validation of actual deposited dose after inhalation

**Figure 1:**
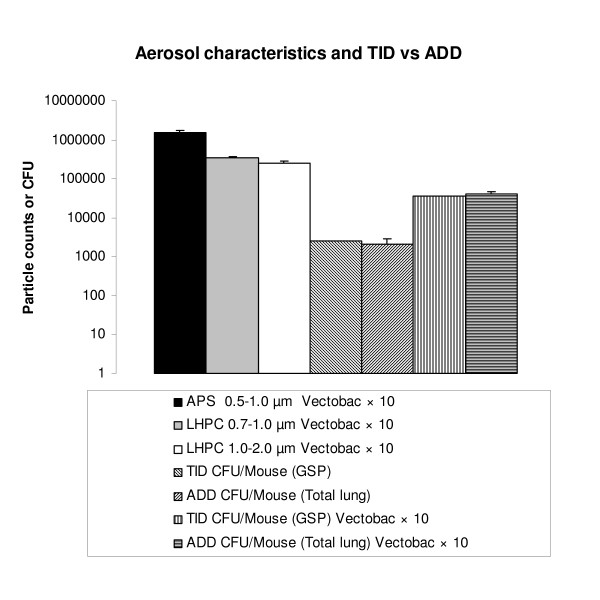
**Aerosol characteristics and validation of actual deposited dose (ADD) per mouse**. Particles (counts min^-1^) of the Vectobac^® ^× 10 exposure aerosol were measured by APS (n= 21) and LHPC (n = 24) for different particle sizes. The theoretically inhaled dose (TID) per mouse based on CFU measurements from a GSP filter sampler were compared to the ADD per mouse (n = 5 per group) for the two different exposure concentrations. Values are means with SEM.

Comparing the theoretically inhaled dose of Vectobac^® ^(3.5 × 10^4 ^CFU) and actual deposited dose (2.9 × 10^4 ^CFU) revealed that 83% of the theoretically inhaled dose was deposited. For the 10 × higher concentration, the mean theoretically inhaled dose was 5.6 × 10^5 ^CFU and actual deposited dose was 5.1 × 10^5 ^CFU, *i.e*. 91% was deposited. The particle counts from APS and LHPC particle counters were stable throughout the exposure (Figure 1).

### CFU recovery from BAL fluid and from total lung homogenate

Comparison of the CFU present in total lung homogenate to the CFU recovered from BAL fluid revealed that an average of 13% (range 10-20%) of the total CFU was recovered by the BAL procedure. The remaining 80-90% of the CFUs were recovered from the lung homogenate of the flushed lungs.

### Acute inflammatory response to biopesticide instillation

**Figure 2:**
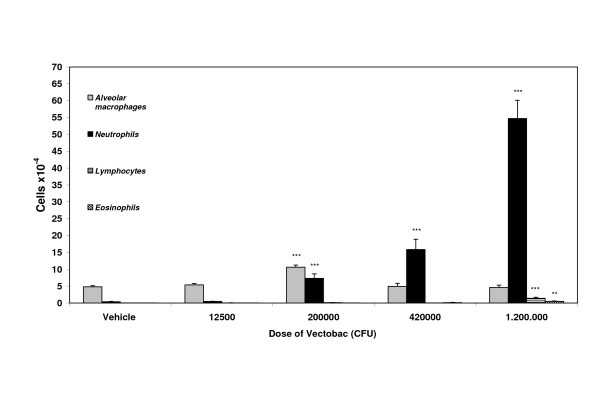
**Cells in BAL fluid after instillation of different doses of biopesticide**. Mean number of cells in bronchoalveolar lavage (BAL) fluid from mice (n = 5 per group) 24 hours after intratracheal instillation of Vectobac^® ^biopesticide. Sterile water served as vehicle and was used for dilutions. For each mouse, 200 cells were counted and differentiated. Values are means with SEM.

A clear dose-dependent increase in number of neutrophils was apparent 24 hours post i.t. instillation of the biopesticide Vectobac^®^. Statistically significant increased numbers of neutrophils were seen after instillation of 2 × 10^5 ^CFU or more. Furthermore, at the 1.2 × 10^6 ^CFU Vectobac^®^dose a significant increased number of lymphocytes and eosinophils were seen (Figure 2).

**Figure 3:**
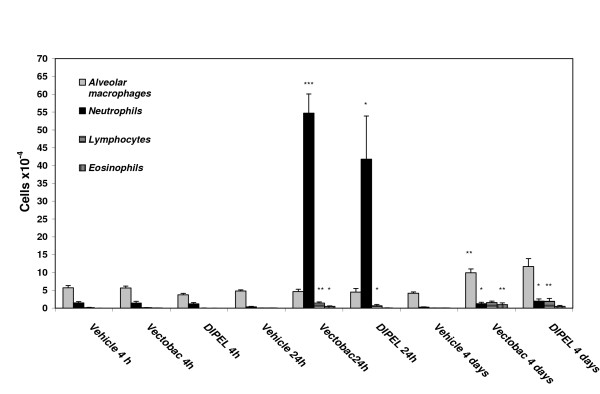
**Cells in BAL fluid at different time points after instillation of biopesticide**. Mean number of cells in bronchoalveolar lavage (BAL) fluid from mice (n = 10 per group) 4 hours, 24 hours or 4 days after intratracheal instillation of Vectobac^® ^or Dipel^® ^biopesticide. Instilled doses of biopesticide were 3.4 × 10^6 ^CFU/mouse for Vectobac^® ^and 3.5 × 10^5 ^CFU/mouse for Dipel^®^. Sterile water served as vehicle and was used for dilutions. For each mouse, 200 cells were counted and differentiated. Values are means with SEM.

The inflammatory responses seen as neutrophils in BALF due to Vectobac^® ^and Dipel^® ^exposures were similar over time as apparent from (Figure 3). No change in cell count or distribution was observed 4 hours after instillation compared to that of the vehicle (sterile water) control groups, but 24 hours post exposure, a significantly increased number of neutrophils were observed for Dipel^® ^(p = 0.03) as well as Vectobac^® ^(p = 0.0001). Four days after exposure, elevated numbers of macrophages and neutrophils were seen for both Dipel^® ^and Vectobac^®^. Furthermore, exposure to Vectobac^® ^gave rise to an increased number of eosinophils (Figure 3).

### Assessment of acute airway irritation after exposure to biopesticide aerosols

For both Vectobac^® ^and Dipel^®^, nine mice were exposed to aerosolised product in the head-only exposure chamber. The aerosols were monitored for both particle counts by LHPC and for size-distribution by APS. The majority of the particles in the generated aerosol were between 0.8 and 2.0 μm with a peak count at 1 μm, which is equal to the size of Bt spores [25]. Each mouse received a theoretically inhaled dose of 1.9 × 10^4 ^CFU Bt *israelensis *or 2.3 × 10^3 ^CFU Bt *kurstaki *per exposure. Respiratory parameters were collected during the first 60 min of exposure to assess airway irritation. The results showed no alterations in respiratory rate, time of brake or time of pause when compared to baseline levels, *i.e*. airway irritation was apparent neither from the nose nor from the lungs (data not shown).

### Recovery of CFU from the sub-chronic (70 days) inhalation and aerosol studies

**Figure 4:**
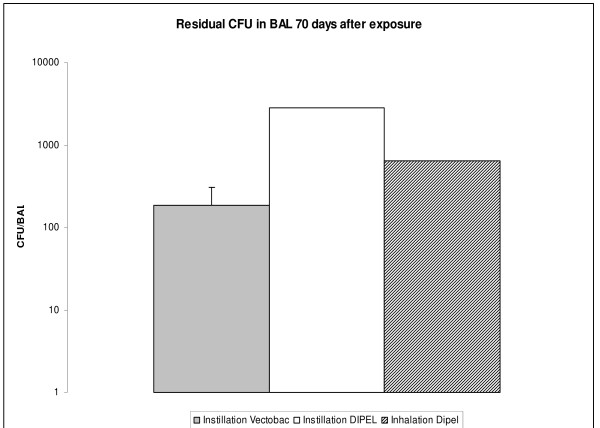
**Number of residual CFU recovered from BAL fluid 70 days after instillation**. For Vectobac^® ^the value is mean with SEM for the responding mice (8 out of 10). For Dipel^® ^instillation or Dipel^® ^inhalation, data represent residual CFU from 1 out of 9 and 1 out of 10 mice, respectively.

All BAL fluids from the sub-chronic studies were also subjected to a CFU count (Figure 4). In the mice instilled with 3.4 × 10^6 ^CFU Vectobac^® ^(8 of 10 mice) bacteria were still present in the BALF with an average of 150 CFU/BALF. Only one mouse out of 9 instilled with 3.5 × 10^5 ^CFU Dipel^® ^had CFU recovered after 70 days (2850 CFU/BALF). In the mice exposed by inhalation to Dipel^® ^aerosols, one mouse out of 10 had CFU recovered (630 CFU/BALF). No CFU was recovered from mice exposed to Vectobac^® ^aerosol.

### Histopathology from the sub-chronic (70 days) studies (experiments 5 and 6)

#### Effects of i.t. instillation

**Figure 5:**
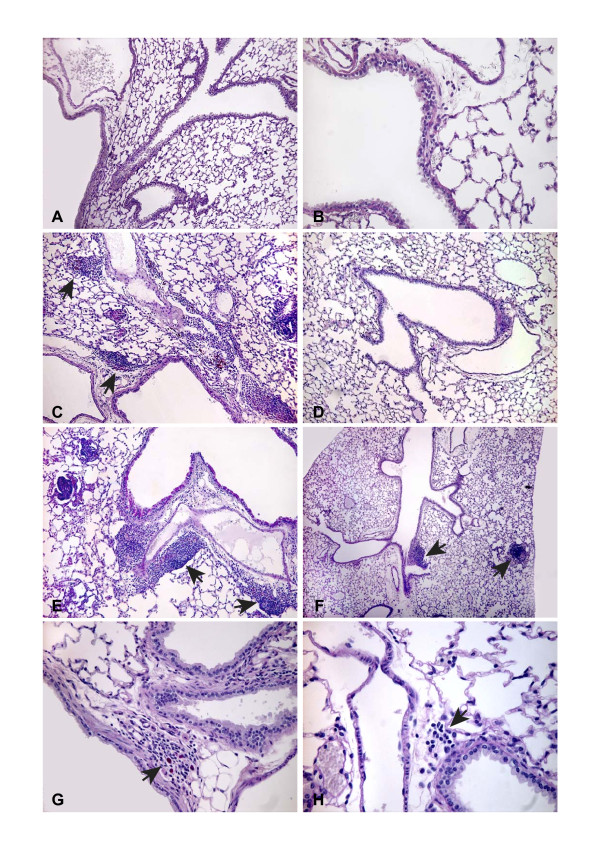
**Lung histology sections from mice 70 days after exposure to biopesticide**. Arrows indicate interstitial inflammation with PAS positive foreign materials. Exposures were 50 μL of sterile pyrogen-free water (Controls), Vectobac^® ^or Dipel^® ^through a single intratracheal instillation (A-F) or repeated (2 × 5 × 1 h) aerosol exposures (G-H). Control slides (A-B) show the pulmonalis and bronchiole wall and with no inflammatory changes. Interstitial inflammation is apparent after Vectobac^® ^instillation (C-E) as indicated by arrows. Instillation of Dipel^® ^resulted in small focal areas with accumulation of inflammatory cells interstitially and inflammation was observed also peripherally even to the level of the pleura (F). Patches of interstitial inflammation were also observed in 3 out of 17 mice after repeated aerosol exposures to Vectobac^® ^(G-H). Sections are stained with periodic acid-Schiff (PAS). Magnifications were ×32 (F), ×80 (A, C, D, E), ×200 (B, G) or ×320 (H).

All 20 mice that received high doses of biopesticide by i.t. instillation showed tissue changes for both commercial products 70 days after exposure. The most pronounced changes were observed in the group given Vectobac^®^. The changes were localized in focal areas adjacent to the larger blood vessels. The dominating cell type was lymphocytes but also plenty of neutrophils and macrophages containing particles were present. The PAS positive material is unidentified material from the biopesticide remaining in the lungs. The sub-chronic inflammation was apparent as small patches of interstitial inflammation, affecting approximately 5% of the lung surface. The degree of inflammation varied considerably within the lung with the most pronounced changes being localized to the lower, posterior part of the lung and only minor changes were observed in the peripheral parts of the lung tissue. Slight interstitial inflammation was observed after Vectobac^® ^instillation (Figures 5C-E). In the larger bronchi, goblet cell formations comparable to experimental bronchitis was observed.

Instillation of Dipel^® ^resulted in fewer and less intense changes. The typical changes were small focal areas with accumulation of inflammatory cells interstitially and inflammation was observed also peripherally even to the level of the pleura (Figure 5F).

#### Effects of aerosol exposure

Histology suggested that one mouse had developed leukaemia. In consequence, data from this mouse was excluded from further analyses. In 3 of the remaining 17 mice, some patches of interstitial inflammation were observed 70 days after end of the repeated exposures to Vectobac^® ^(Figure 5G and 5H), whereas exposure to Dipel^® ^gave rise to less significant effects (not shown).

## Discussion

The Bt based biopesticides are generally considered a safe and greener alternative to chemical pesticides. The commercial Bt species are believed to be non-infectious and have only on rare occasions been associated with opportunistic infections in humans. Nevertheless, the close relationship between Bt and the human pathogen *Bacillus cereus *continues to be substantiated and gives rise to new questions [26-29].

The present study showed that instilled or even inhaled Bt spores may be present in the lung and extracted by BAL 70 days after administration. Our data are in line with other clearance studies, demonstrating CFU of *Bt kurstaki *in the liver, spleen and lungs 21 days after intratracheal (i.t.) instillation and similar patterns were seen with *Bt aizawai *and *B. subtilis*. Clearance patterns after i.v. injection with 10^7 ^CFU per animal is also reported for *Bt kurstaki, Bt israelensis, B. subtilis *and *B. sphaericus*. All strains were still recovered from inner organs at the termination of the study (day 57 for *Bt israelensis *and 128 for *Bt kurstaki*) [30,31].

As Bt formulations are used for spray application, hazard identification and risk assessment should be based on airway effects. To our knowledge, the present study is the first to investigate airway irritation and airway inflammation induced by inhalation of commercial Bt biopesticides. The i.t. instillation of biopesticide, showed that a single exposure gave rise to focal areas of lung tissue inflammation still detectable 70 days after exposure. A clear dose-response relationship was seen. Inflammation was also seen 70 days after repeated inhalation of Bt biopesticide, although the effects after inhalation were less vigorous than after instillation. The sub-chronic inflammation was apparent as small patches of interstitial inflammation, a response that was not detectable in the corresponding BAL fluid. The sub-chronic inflammation observed in the present study, was most likely due to the prolonged presence of Bt spores or other product residues in the lungs, triggering and maintaining the inflammatory response. This should be seen in the light that the formulated biopesticides contains only about 2% spores and 98% other ingredients according to manufacturer which makes long term inhalation studies using the final formulated biopesticide important. The list of other ingredients besides water is known to authorities (e.g. the EPA) and approved for other purposes e.g. a "food- carbohydrate" and preservatives [32]. Most of these other ingredients have probably not been subjected to long term inhalation studies in animals as this was not their intended use. Therefore alternative inoculums or controls, including spore free or heat-inactivated biopesticide or specific excipients/additives should also be studied for biological effect. In the case of low clearance rates, as demonstrated in this study, the inflammation could be prolonged or even become chronic which may potentially initiate the development of severe health effects such as chronic obstructive pulmonary disease [33] or formation of fibrotic lung tissue [34].

In experiments 3, 5 and 6 the exposure concentrations of Dipel^® ^were almost a 10-fold lower than Vectobac^® ^and the lower effects and tissue changes of the exposures with Dipel^® ^should be seen in this light. This difference is also shown as the recovery of CFU still present in the BAL fluids 70 days after instillation with different inoculums of two biopesticides. The lower concentrations were chosen on the basis of experiment 4, where a washing procedure of the Dipel^® ^product was necessary due to viscosity. A pilot experiment revealed that the washing procedure did not change the inflammatory properties of the product. Upon dilution of the Dipel^®^, the viscosity was acceptable for instillation, wherefore suspensions of the unaltered commercial Dipel^® ^product were used.

Our study has also demonstrated that exposure to aerosolized Vectobac^® ^did not induce airway irritation upon inhalation. This is important in regards to occupational hazard as the absence of discomfort by exposure would make workers less inclined to wear the recommended protective filter facemask while working with the biopesticide.

## Conclusions

Repeated exposure to biopesticide aerosols may lead to sub-chronic lung inflammation which may contribute to the development of severe lung diseases. No airway irritation was observed upon inhalation of Bt aerosols, suggesting that exposure will not evoke a warning signal, making the exposure insidious.

The present study emphasises the need for additional studies assessing lung effects after long-term, repeated exposures to low and occupationally relevant concentrations of Bt biopesticide aerosols.

## List of abbreviations used

APS: Aerodynamic particle sizer; ADD: Actual deposited dose; BAL(F): Bronchoalveolar lavage (fluids); BCSA: *Bacillus cereus *selective agar; Bt: *Bacillus thuringiensis*; CFU: Colony forming units; COPD: Chronic obstructive pulmonary disease; GSP: Gesamtstaubprobenahme sampler; I.t.: Intra tracheal; LHPC: Lighthouse handheld particle counter; NOEL: No-observed effect level; PAS: Periodic acid Schiff; TID: Theoretically inhaled dose

## Authors' contributions

KKB, MHA and STL designed the studies and planned the experiments. KKB, MHA and SSP conducted the laboratory work. KKB, SSP and STL interpreted the data. KKB drafted the first version of the manuscript. All authors contributed to and approved the final manuscript.
